# Molecular mechanisms of autophagy-lysosomal pathway dysfunction in neurodegenerative diseases and therapeutic strategies for lysosomal repair: a review

**DOI:** 10.3389/fnins.2026.1819002

**Published:** 2026-05-15

**Authors:** Weilin Wang, Xianguo Meng

**Affiliations:** 1 Medical Science and Technology Innovation Center, Shandong First Medical University and Shandong Academy of Medical Sciences, Jinan, Shandong, China; 2 Department of Rehabilitation Medicine, The First Affiliated Hospital of Shandong First Medical University & Shandong Provincial Qianfoshan Hospital, Jinan, Shandong, China; 3 Shandong First Medical University, Jinan, Shandong, China

**Keywords:** autophagy-lysosomal pathway, lysosomal repair, molecular mechanisms, neurodegenerative diseases, protein degradation, therapeutic strategies

## Abstract

The autophagy-lysosomal pathway (ALP) is a critical intracellular protein degradation system responsible for maintaining proteostasis and metabolic balance within cells. Dysfunction of this pathway has been increasingly recognized as a key pathological basis underlying various neurodegenerative diseases (NDs). This review provides a comprehensive overview of the molecular mechanisms by which ALP impairment contributes to defective protein degradation in neurodegeneration. We focus on the impact of lysosomal structural integrity and functional imbalance on cellular fate, highlighting the interplay between protein oxidative damage and degradation system dysregulation. Furthermore, we summarize the current therapeutic strategies aimed at lysosomal repair, evaluating their potential clinical applications and efficacy. By integrating the latest research advances, this review aims to deepen the understanding of the pathological mechanisms of autophagy-lysosomal pathway dysfunction in neurodegenerative diseases, clarify the key molecular targets of lysosomal damage and repair, and provide theoretical basis for target screening and validation and practical reference for the development of targeted drugs for neurodegenerative diseases.

## 1 Introduction

Neurodegenerative diseases (NDs), including Alzheimer’s disease (AD), Parkinson’s disease (PD), and amyotrop9ic lateral sclerosis (ALS), represent a significant challenge for modern medicine due to their complex etiology and progressive nature (Fereshtehnejad and Lökk, 2025; Gadhave et al., 2024; Liu and Song, 2025; Mohamed Yusoff and Mohd Khair, 2024; Sanghai and Tranmer, 2023). Characterized by the accumulation of misfolded proteins and cellular dysfunction, these disorders are closely associated with the impairment of the ALP, which is critical for maintaining cellular homeostasis and protein quality control (Fereshtehnejad and Lökk, 2025; Gadhave et al., 2024; Liu and Song, 2025; Mohamed Yusoff and Mohd Khair, 2024; Sanghai and Tranmer, 2023).

For example, lysosomal storage diseases (LSDs) are abnormal accumulation of substances in the lysosomes of affected cells, eventually leading to neurodegenerative diseases (Perez et al., 2020). The autophagy-lysosomal system is responsible for degrading damaged organelles and misfolded proteins, thus preventing their toxic accumulation (Nixon and Rubinsztein, 2024). Dysfunction within this pathway is increasingly recognized as a central mechanism driving neurodegeneration, leading to neuronal death and cognitive decline (Lee et al., 2024).

The autophagy-lysosomal pathway (ALP) involves various cellular processes, including the formation of autophagosomes, fusion with lysosomes, and subsequent degradation of cellular components (Yu et al., 2018; Zhi et al., 2018). Recent research has highlighted the intricate relationship between lysosomal dysfunction and NDs (Nixon and Rubinsztein, 2024; Nixon, 2020). For instance, LSDs, characterized by the accumulation of undegraded substrates due to enzymatic deficiencies, often manifest severe neurological symptoms, underscoring the critical role of lysosomal integrity in brain homeostasis (Hyun and Lee, 2021). Moreover, genetic mutations affecting lysosomal proteins have been implicated in both familial and sporadic cases of NDs, further emphasizing the role of lysosomal dysfunction in these conditions (Navarro-Romero et al., 2020).

Lysosomes are not merely waste disposal units; they serve as signaling hubs that integrate nutrient sensing and metabolic regulation (Lamming and Bar-Peled, 2019; Mony et al., 2016). Their dysfunction can lead to a cascade of cellular events, including oxidative stress, inflammation, and impaired neuronal function (Quick et al., 2023; Saipuljumri et al., 2026). For example, recent studies have shown that lysosomal acidification is crucial for optimal enzyme activity and substrate degradation, and that disruptions in lysosomal pH can exacerbate neurodegenerative processes (Lo and Zeng, 2023). This highlights the need to better understand the molecular mechanisms underlying lysosomal dysfunction and its contributions to neurodegenerative pathology.

A growing body of evidence suggests that enhancing lysosomal function may offer therapeutic opportunities for treating NDs (Mercer et al., 2025; Udayar et al., 2022). Strategies aimed at restoring lysosomal integrity, such as pharmacological agents that promote autophagy or enhance lysosomal biogenesis, have shown promise in preclinical models (Fang et al., 2026). Furthermore, the advent of novel therapeutic modalities, such as proteolysis-targeting chimeras (PROTACs) and lysosome-targeting compounds, presents exciting possibilities for selectively degrading toxic protein aggregates associated with neurodegeneration (Krogsaeter et al., 2022). For instance, the blocker of the synthetic mucin TRP channel 1 (TRPML1) can inhibit lysosomal particle uptake and lysosomal exocytosis, while the use of TRPML1 agonists can reverse these phenomena, thereby eliminating senescent and apoptotic cells *in vivo* (Samie et al., 2013).

In conclusion, ALP plays a pivotal role in the pathogenesis of NDs. Understanding the molecular mechanisms that govern lysosomal function and their dysregulation in neurodegenerative contexts is essential for developing effective therapeutic strategies. This review aims to explore the intricate relationship between autophagy-lysosomal dysfunction and neurodegeneration, highlighting potential avenues for intervention and the therapeutic implications of restoring lysosomal health in the treatment of these devastating disorders.

## 2 Biological functions and structural features of the autophagy-lysosome pathway

**FIGURE 1:**
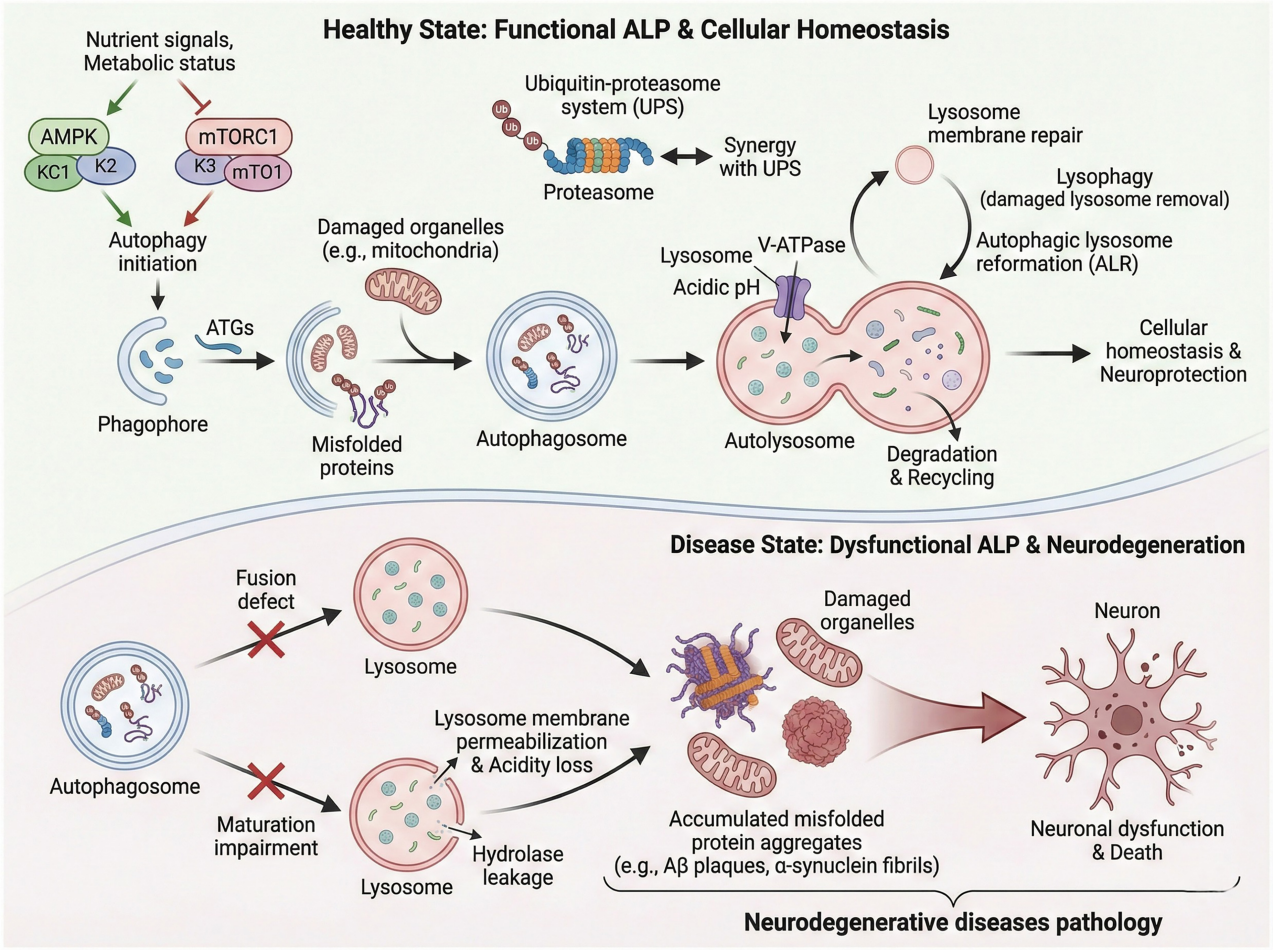
Schematic diagram of the functional differences of autophagy-lysosomal pathway (ALP) under physiological and pathological conditions. Under physiological conditions, the ALP maintains cellular proteostasis and metabolic balance through sequential processes including autophagosome biogenesis, fusion with lysosomes, and degradation of damaged organelles/misfolded proteins (upper panel). Key regulatory molecules (e.g., mTORC1, AMPK, TFEB) and synergistic interactions with the ubiquitin-proteasome system (UPS) are involved in this process. Under pathological conditions [e.g., neurodegenerative diseases (NDs)], ALP dysfunction is characterized by impaired autophagosome-lysosome fusion, lysosomal membrane permeabilization (LMP), and reduced degradation capacity, leading to the accumulation of toxic protein aggregates (e.g., amyloid-beta, alpha-synuclein, TDP-43) and ultimately neuronal damage or death (lower panel).

The ALP constitutes a core cellular catabolic and homeostatic machinery, evolutionarily conserved across eukaryotes and indispensable for maintaining intracellular proteostasis, organelle quality control, and metabolic adaptation (Gómez-Virgilio et al., 2022; Lippai and Szatmári, 2017; Ryter et al., 2019; Yamamoto et al., 2023; Yu et al., 2018). Beyond its basal role in degrading damaged cellular components and recycling bioactive molecules to sustain normal cellular physiology, the ALP exerts context-dependent regulatory effects on ALP-related cell death decisions, immune modulation, and stress responses, with its functional integrity tightly linked to the structural stability of lysosomes and their synergistic crosstalk with other protein degradation systems such as the ubiquitin-proteasome system (UPS) (Elsasser et al., 2023; Mahapatra et al., 2021; Nam et al., 2017). Dysregulation of the ALP—including impaired autophagosome biogenesis and maturation, lysosomal membrane permeabilization (LMP), defective intersystem crosstalk, and disrupted lysosomal signaling—has emerged as a pivotal pathological driver in a spectrum of human diseases, most notably neurodegenerative disorders, where the progressive loss of ALP function underpins the accumulation of toxic protein aggregates, neuronal dysfunction, and irreversible cell death (Lee et al., 2022; Malik et al., 2019; Nixon and Rubinsztein, 2024; Zhao et al., 2021). In this section, we systematically elaborate the canonical process of autophagy and its function in protein degradation, as well as the structural and functional regulation of lysosomes. We also summarize the synergistic action of intracellular protein degradation systems, the regulatory role of ALP in cell fate, and the typical abnormal manifestations of ALP in neurodegenerative diseases. These contents lay a foundational framework for the following discussion on pathological mechanisms and therapeutic targets of this pathway (Figure 1).

### 2.1 The process of autophagy and its role in protein degradation

Autophagy is a highly regulated and evolutionarily conserved cellular process that plays a critical role in maintaining cellular homeostasis by degrading and recycling cellular components (Mizushima and Komatsu, 2011; Zhu et al., 2022). This process begins with the formation of a double-membrane structure known as the autophagosome, which encapsulates various cellular debris, including damaged organelles, misfolded proteins, and other waste materials (Yorimitsu and Klionsky, 2005). Following this encapsulation, the autophagosome fuses with lysosomes, leading to the degradation of its contents by lysosomal enzymes (Awan and Deng, 2014). This fusion is a crucial step in autophagy, as it allows for the breakdown of the encapsulated materials into smaller molecules that can be recycled and used by the cell for energy or as building blocks for new cellular components (Shao et al., 2025). The regulation of autophagy is intricate, involving numerous autophagy-related genes (ATGs) that orchestrate the formation and maturation of autophagosomes. These genes ensure that the autophagic process is efficient and selective, thereby facilitating the effective clearance of intracellular proteins and damaged organelles (Ghosh and Ranjan, 2022).

Moreover, autophagy is not merely a catabolic process, it also plays a significant role in various cellular functions, including metabolic regulation, stress responses, and immune modulation (Deretic, 2021; White et al., 2021). Under normal conditions, autophagy operates at basal levels to remove damaged cellular components and maintain metabolic equilibrium. However, during stress conditions, such as nutrient deprivation or oxidative stress, autophagy is upregulated to enhance cellular survival by providing essential nutrients through the recycling of cellular components (Cozzi and Ferrari, 2022; Jahreiss et al., 2008). This dynamic nature of autophagy highlights its importance in adapting to changing cellular environments and stresses. Furthermore, autophagy has been implicated in various physiological and pathological processes, including NDs, where the accumulation of misfolded proteins can overwhelm the cellular degradation systems, leading to cellular dysfunction and death (Ghosh and Ranjan, 2022).

The involvement of autophagy in the degradation of cellular fibrillar deposits is particularly noteworthy, as it serves as a key protective mechanism against proteotoxic stress. For instance, in NDs like Alzheimer’s and Parkinson’s, impaired autophagy can lead to the accumulation of toxic protein aggregates, exacerbating neuronal damage (Hayes, 2019; Lane et al., 2018; Weller and Budson, 2018; Zhang et al., 2024). Recent studies have shown that enhancing autophagic activity can promote the clearance of these aggregates, suggesting that autophagy-targeting therapies may hold therapeutic potential for treating neurodegenerative conditions (Ding et al., 2022; Fu et al., 2025). Additionally, the selective degradation of specific proteins via autophagy has opened new avenues for targeted protein degradation strategies in drug development, where compounds can be designed to harness the autophagy machinery for the elimination of disease-causing proteins (Ji et al., 2022b).

In conclusion, autophagy is a crucial cellular process that not only facilitates the degradation of proteins and organelles but also plays a vital role in maintaining cellular homeostasis and responding to stress. Understanding the mechanisms that regulate autophagy and its implications in various diseases is essential for developing novel therapeutic strategies aimed at enhancing autophagic function and improving cellular health. As research continues to unveil the complexities of autophagy, it is clear that this process represents a promising target for therapeutic interventions in a range of diseases characterized by protein aggregation and cellular dysfunction.

### 2.2 Endosomal maturation failure and its pathogenic mechanisms

The proper functioning of the lysosomal system is intrinsically dependent on the upstream maturation of endosomes. The endo-lysosomal pathway involves the internalization of cargo into early endosomes, which then undergo a critical maturation process to become late endosomes and multivesicular bodies (MVBs) before eventually fusing with lysosomes for degradation (Todd et al., 2023). This transition is primarily governed by a “Rab switch,” where Rab5-positive early endosomes mature into Rab7-positive late endosomes, a process synchronized with the conversion of phosphoinositide PI3P to PI(3,5)P2 by the kinase PIKfyve (Todd et al., 2023).

Recent evidence highlights that failures at these maturation stages are central to the pathogenesis of ALS/FTD and other NDs. For instance, mutations in Vps54 (as seen in the wobbler mouse model) destabilize the GARP complex, leading to impaired retrograde transport and the accumulation of enlarged, dysfunctional Rab7-positive late endosomes (Palmisano et al., 2011; Schmitt-John et al., 2005). Similarly, TBK1 deficiency disrupts the phosphorylation of Rab7, thereby stalling endosomal maturation and facilitating the development of TDP-43 proteinopath (Talaia et al., 2024). The ESCRT-III complex, involving CHMP2B, is also essential for sorting cargo into MVBs; mutations in CHMP2B result in Rab7 recruitment failure and inefficient endosomal-mediated degradation (Clayton et al., 2018; Urwin et al., 2010). Notably, disrupting endosomal maturation—either by inhibiting PIKfyve or overexpressing active Rab5—has been shown to be sufficient to trigger endogenous TDP-43 aggregation, even when terminal autophagic clearance is not yet fully compromised (Shao et al., 2022; Talaia et al., 2024). These findings underscore that endosomal maturation failure is an independent and early pathogenic event in ALP failure, directly contributing to the accumulation of toxic protein aggregates.

### 2.3 Structural integrity of lysosomes and their functional regulation

Lysosomes are critical organelles within cells, acting as the primary digestive centers responsible for the degradation of various macromolecules, including proteins, lipids, and nucleic acids (Trivedi et al., 2020). Their functionality is heavily reliant on maintaining an acidic internal environment, which is essential for the optimal activity of the numerous hydrolytic enzymes they contain (Appelqvist et al., 2013; Yim and Mizushima, 2020). This acidic milieu is achieved through the action of the vacuolar-type H+-ATPase (V-ATPase), which pumps protons into the lysosomal lumen, thus maintaining the necessary pH for enzymatic activity (Mahendran et al., 2025). The structural integrity of lysosomes is paramount; any disruption can lead to LMP, resulting in the leakage of hydrolytic enzymes into the cytosol, which can trigger cellular apoptosis or inflammation (Kavčič et al., 2017; Serrano-Puebla and Boya, 2016). Recent studies have elucidated various mechanisms that cells employ to repair damaged lysosomes, including lysophagy, where damaged lysosomes are selectively degraded and replaced, ensuring that the organelles maintain their structural and functional integrity (Tsujimoto et al., 2023).

Beyond their role in degradation, lysosomes also function as signaling platforms that integrate various metabolic signals (Inpanathan and Botelho, 2019). They are involved in the regulation of critical signaling pathways, such as the mechanistic target of rapamycin complex 1 (mTORC1) and AMP-activated protein kinase (AMPK) pathways, which are essential for cellular metabolism and energy sensing (Bi et al., 2025). The Ragulator complex, located on the lysosomal membrane, plays a pivotal role in tethering mTORC1 to lysosomes, facilitating its activation in response to nutrient availability. This interaction underscores the importance of lysosomal integrity not only for degradation processes but also for signaling pathways that govern cellular homeostasis and metabolic adaptation (Tsujimoto et al., 2023).

Lysosomal quality control (LQC) mechanisms are crucial for maintaining the dynamic balance of lysosomal structure and function (Chauhan and Patro, 2024; Yang and Tan, 2023). These mechanisms include lysosomal repair processes, lysophagy, and the regeneration of lysosomes through autophagic pathways (Chauhan and Patro, 2024; Ferrari et al., 2024; Henn et al., 2025; Nanayakkara et al., 2023; Yang and Tan, 2023). For instance, when lysosomes are damaged, they can undergo a repair process that involves the recruitment of specific proteins that stabilize the lysosomal membrane and restore its function (Zhong and Richardson, 2025). Additionally, the autophagic lysosome reformation (ALR) pathway plays a significant role in replenishing lysosomal populations after degradation, ensuring that cells can continue to efficiently manage waste and maintain metabolic balance (McGrath et al., 2021). This interplay between lysosomal integrity, signaling, and quality control is vital for cellular health and has implications for various diseases, particularly neurodegenerative disorders where lysosomal dysfunction is a hallmark (Zhong and Richardson, 2025).

In summary, the structural integrity of lysosomes and their functional regulation are interlinked through complex mechanisms that ensure cellular homeostasis. The maintenance of an acidic environment, the integration of signaling pathways, and the deployment of quality control mechanisms collectively underscore the importance of lysosomes in cellular physiology. Understanding these processes is crucial, as dysregulation can lead to a range of pathological conditions, including neurodegeneration and metabolic disorders. The ongoing research into lysosomal function and integrity holds promise for future therapy development.

### 2.4 The synergy between ALP and protein degradation systems

The interplay between the ALP and the 20S proteasome system is crucial for maintaining cellular protein homeostasis, particularly in the context of NDs (Bustamante et al., 2018). Both systems serve as essential mechanisms for degrading damaged and misfolded proteins, thereby preventing the accumulation of toxic aggregates that can lead to cellular dysfunction (Bustamante et al., 2018; Selvaraj et al., 2025). The ALP is responsible for the bulk degradation of cytoplasmic components, including long-lived proteins and organelles, while the proteasome primarily targets short-lived proteins and ubiquitinated substrates for degradation (Dikic, 2017). Research has demonstrated that these two systems are not only complementary but also synergistically enhance each other’s efficiency in maintaining proteostasis (Bustamante et al., 2018). For instance, in conditions where one pathway is impaired, the other can compensate to some extent, although this compensation is often insufficient to fully restore cellular homeostasis (Ji et al., 2022b). The balance between autophagic and proteasomal degradation is particularly critical in neurons, which are post-mitotic and cannot dilute the burden of misfolded proteins through cell division (Cai and Ganesan, 2022). Consequently, disruptions in either pathway can lead to the accumulation of toxic protein aggregates, contributing to the pathogenesis of neurodegenerative disorders such as Alzheimer’s and PD (Romano et al., 2025).

Moreover, the degradation burden increases significantly in the presence of oxidative stress, which is a common feature in NDs (Kinger et al., 2024; Wilson et al., 2023). Oxidative damage leads to the formation of oxidized proteins that are more challenging to degrade, necessitating a highly efficient and coordinated response from both the autophagy and proteasome systems (Kinger et al., 2024; Kocaturk and Gozuacik, 2018). The accumulation of oxidatively modified proteins can overwhelm the proteasomal capacity, thereby shifting the degradation load toward the autophagy-lysosome pathway. This shift is critical for maintaining cellular health, as it allows for the clearance of larger aggregates that the proteasome cannot handle effectively. In this context, the ability of the ALP to adapt and respond to increased protein degradation demands underscores its importance in cellular quality control mechanisms (Ji et al., 2022a).

Additionally, recent advancements in targeted protein degradation technologies, such as autophagy-targeting chimeras (AUTACs) and PROTACs, have highlighted the potential of harnessing the synergistic action of these two degradation pathways for therapeutic purposes (Sandhof et al., 2025). By designing molecules that can selectively direct misfolded or aggregation-prone proteins to either the proteasome or the autophagy-lysosome system, researchers are exploring innovative strategies to enhance protein clearance in NDs (Liu P. et al., 2022). Such approaches not only aim to alleviate the burden of toxic protein aggregates but also to restore the balance between the two degradation systems, thereby improving cellular resilience against proteotoxic stress (Sandhof et al., 2025). Although the synergy between ALP and the UPS is well-documented, we contend that most studies have focused on acute inhibition of one pathway while observing compensatory activation of the other. Chronic disease conditions, however, likely involve a more complex, time-dependent rewiring of degradation networks. A key unanswered question is whether restoring one pathway can fully compensate for the other when dysfunction persists over months or years-a scenario more relevant to human neurodegeneration.

### 2.5 ALP and aLP-related cell death regulation

The ALP plays a pivotal role in regulating ALP-related cell death, particularly in the context of NDs where it is often disrupted (Nixon and Rubinsztein, 2024). When lysosomal integrity is compromised, it can lead to the leakage of lysosomal enzymes into the cytosol, triggering various forms of cell death, including apoptosis, necrosis, and ferroptosis (Liu et al., 2023). This leakage not only initiates a cascade of cellular events that culminate in cell death but also contributes to the pathological progression of NDs (Mançano et al., 2024). The dysfunction of lysosomes leads to the aggregation of amyloid proteins related to the pathogenesis of Alzheimer’s disease (AD), but the permeabilization and subsequent rupture of lysosomes also participate in the pathological process of AD. In the early stage of AD, the levels of lysosomal activation proteins (SAPs) and lysosome-associated membrane protein 1 (LAMP1) around the amyloid plaques increase (Sharoar et al., 2021), which may cause lysosomal membrane leakage, thereby releasing membrane components (such as proteases) into the cytoplasm, and subsequently triggering lysosome-dependent cell death, which is one of the reasons for the early loss of neurons in AD (Wang et al., 2018). For example, cytoplasmic tissue protease B (CSTB) can initiate a protein hydrolysis cascade reaction, by degrading anti-apoptotic protein Bcl-xl and activating pro-apoptotic protein Bid, ultimately leading to cell apoptosis (de Castro et al., 2016). In addition, the release of tissue proteases can trigger neuroinflammation by inducing the release of inflammatory factors from microglia. For example, tissue protease C can induce microglial M1 polarization by activating the Ca^2+^-dependent PKC/p38MAPK/NF-κB pathway. This further exacerbates neuroinflammation (Liu et al., 2019). The maintenance of lysosomal integrity is thus critical for neuronal survival, as it prevents the toxic accumulation of cellular debris and mitigates the risk of cell death (Kim et al., 2025).

Moreover, the interplay between lysosomal function and cellular metabolism is vital for neuronal health (Lie and Nixon, 2019). Dysregulation of ALP can lead to metabolic imbalances that affect neuronal viability (Kim et al., 2023). Research indicates that lysosomal dysfunction is closely linked to metabolic disorders, which in turn can influence neuronal survival. For instance, impaired autophagic flux can result in the accumulation of dysfunctional organelles and toxic metabolites, ultimately leading to cell death (Tegeder and Kögel, 2021). Conversely, proper autophagic activity is essential for the removal of damaged components, allowing for metabolic adaptation and promoting cell survival under stress conditions (White et al., 2021). This dynamic balance underscores the importance of the ALP in determining ALP-related cell death, especially in the context of NDs where metabolic dysregulation is a common feature (Finkbeiner, 2020).

Furthermore, the relationship between lysosomal dysfunction and autophagy is complex and context-dependent. In some instances, excessive autophagy can lead to autophagic cell death, a phenomenon observed in various pathological states. For example, in certain cancer cells, the activation of autophagy can provide a survival advantage under stress; however, when dysregulated, it can trigger cell death (Du et al., 2025). This dual role of autophagy highlights the necessity of tightly regulating lysosomal function and autophagic processes to maintain cellular homeostasis and prevent pathological outcomes. The disruption of this balance can have dire consequences, particularly in neurons, which are highly susceptible to metabolic stress and damage.

In summary, ALP is integral to the regulation of ALP-related cell death, influencing both survival and death mechanisms in neurons. The maintenance of lysosomal integrity is essential for preventing cell death and ensuring metabolic balance, while dysregulation of this pathway can lead to NDs and other pathological conditions. Understanding the molecular mechanisms underlying these processes offers potential therapeutic avenues for targeting lysosomal dysfunction and restoring autophagic balance in NDs and other disorders characterized by impaired cellular homeostasis.

### 2.6 Abnormal manifestations of the autophagy-lysosome pathway in NDs

Given that neurons are post-mitotic and possess elaborate morphologies, they exhibit a heightened sensitivity to ALP dysfunction (Sidibe et al., 2022). In various NDs, a common abnormal manifestation is the impaired fusion between autophagosomes and lysosomes, leading to the accumulation of misfolded proteins and damaged organelles (Nixon and Rubinsztein, 2024), such as α-synuclein in PD, amyloid-β plaques and tau tangles in AD, huntingtin in HD, Tau, TDP-43 and FUS in FTD, and TDP-43, SOD1, and FUS in ALS, is considered a hallmark of neurodegenerative diseases (NDs), including ALS and FTD (Beckers and Van Damme, 2025; Burrell et al., 2016; Soto and Pritzkow, 2018; Wyss-Coray, 2016). The inability of autophagosomes to effectively fuse with lysosomes disrupts the degradation process, causing a backlog of cellular debris that exacerbates neurodegenerative pathology (Tedeschi et al., 2025). Notably, chaperone-mediated autophagy (CMA) may be activated as a compensatory pathway under this condition, which can selectively recognize and degrade abnormally folded proteins, thereby partially alleviating proteotoxicity and providing a potential therapeutic target for neurodegenerative diseases (Bourdenx et al., 2021). Consequently, the disruption of the autophagy-lysosome pathway is not merely a consequence of neurodegeneration but is increasingly recognized as a central mechanism driving the progression of these diseases.

Additionally, the release of key proteins such as TDP-43 through autophagy-dependent extracellular vesicles (EVs) has been implicated in the intercellular propagation of pathological proteins. TDP-43, a protein associated with frontotemporal dementia and amyotrophic lateral sclerosis, is released from affected neurons and taken up by neighboring cells, facilitating the spread of neurodegenerative pathology (Nixon, 2024). This process underscores the importance of autophagy in not only clearing toxic aggregates but also in mediating the communication of pathological states between neurons. The dysregulation of this pathway can lead to a vicious cycle where the accumulation of toxic proteins promotes further autophagic dysfunction, thereby enhancing the spread of neurodegeneration (Zeng et al., 2024).

Moreover, lysosomal dysfunction exacerbates oxidative stress within neurons, creating a detrimental feedback loop that accelerates neurodegenerative processes. The impaired autophagy-lysosome pathway leads to the accumulation of reactive oxygen species (ROS), which can damage cellular components, including lipids, proteins, and DNA (Jiao et al., 2024). This oxidative stress not only contributes to neuronal cell death but also activates inflammatory responses that further compromise neuronal integrity. The interplay between oxidative stress and lysosomal dysfunction highlights the complexity of NDs, where multiple pathological mechanisms converge to drive disease progression (Mishra et al., 2022).

In summary, the abnormal manifestations of the autophagy-lysosome pathway in NDs are characterized by impaired autophagosome-lysosome fusion, the intercellular spread of pathological proteins, and exacerbated oxidative stress. Understanding these mechanisms is crucial for developing targeted therapeutic strategies aimed at restoring autophagic function and mitigating the progression of NDs. Addressing the multifaceted nature of these dysfunctions may provide new avenues for intervention, potentially improving outcomes for patients suffering from these debilitating conditions.

## 3 Molecular mechanisms of protein oxidative damage and dysfunction of autophagy-lysosome pathway

In this section, we systematically review the molecular mechanisms underlying oxidative stress-induced protein damage, the crosstalk between oxidative stress and autophagy-lysosome dysfunction, oxidative modifications of key autophagic proteins, unconventional extracellular release of pathological proteins upon autophagy blockade, as well as the causal link between impaired protein degradation and progressive neuronal dysfunction. Elucidating these interconnected processes will provide novel insights into the pathogenesis of oxidative stress- and autophagy-related disorders and facilitate the development of targeted therapeutic strategies.

### 3.1 The impact of oxidative stress on protein structure and function

Oxidative stress induces irreversible post-translational modifications, such as hydroxylation and carbonylation, which disrupt protein conformation and structural stability (Chiriţoiu et al., 2023; Sultana and Butterfield, 2024; Zavadskiy et al., 2022; Zhang et al., 2020). These ROS-driven modifications lead to functional loss and protein aggregation, ultimately contributing to cellular dysfunction and the pathogenesis of various diseases (Chiriţoiu et al., 2023; Sultana and Butterfield, 2024; Zavadskiy et al., 2022; Zhang et al., 2020).

The aberrant modifications induced by oxidative stress can lead to protein misfolding and aggregation, which are hallmarks of many NDs (Orfali et al., 2024). Proteins that are oxidatively damaged often fail to fold correctly, leading to the formation of aggregates that can disrupt cellular homeostasis and trigger toxic pathways (Del Monte and Agnetti, 2014). For example, in conditions such as AD, the accumulation of misfolded amyloid-beta peptides is exacerbated by oxidative modifications, which impair their clearance and promote further aggregation (Ahmadi et al., 2025). The aggregation of these misfolded proteins can lead to a toxic environment, causing neuronal damage and loss of function (Ochneva et al., 2022). Additionally, the presence of oxidatively modified proteins can overwhelm the cellular degradation systems, such as the UPS and autophagy pathways, which are responsible for maintaining protein homeostasis. This inability to effectively clear damaged proteins contributes to the accumulation of cellular debris and further exacerbates oxidative stress, creating a vicious cycle that accelerates neurodegeneration.

Moreover, proteins that have undergone oxidative damage are often less recognizable to the cellular degradation systems, making them more likely to persist within the cell and exert toxic effects. For example, oxidized proteins may exhibit altered surface properties that hinder their recognition by chaperones and proteolytic complexes, leading to increased cellular toxicity and impaired function (Duong et al., 2024; Grune et al., 2003). This phenomenon is particularly concerning in the context of aging and neurodegenerative diseases, where the accumulation of oxidized proteins correlates with increased cellular stress and dysfunction (Eggers et al., 2025; Korovesis et al., 2023). As a result, understanding the molecular mechanisms underlying oxidative stress and its impact on protein structure and function is crucial for developing therapeutic strategies aimed at mitigating the effects of oxidative damage and restoring cellular homeostasis.

In summary, oxidative stress leads to irreversible modifications of protein residues, resulting in structural and functional impairments. The subsequent misfolding and aggregation of proteins not only compromise their individual functions but also disrupt cellular proteostasis, contributing to the pathogenesis of various diseases, particularly neurodegenerative disorders. Addressing oxidative stress and its consequences on protein integrity may provide valuable insights into potential therapeutic approaches for these conditions.

### 3.2 The imbalance of oxidative stress in regulating ALP

Oxidative stress is a significant factor that disrupts the delicate balance of ALP, leading to impaired cellular homeostasis. ROS are produced as byproducts of normal cellular metabolism but can accumulate excessively under pathological conditions, such as NDs. This accumulation of ROS can interfere with the formation of autophagosomes and the functionality of lysosomes, crucial components of ALP. Specifically, oxidative stress disrupts the fusion process between autophagosomes and lysosomes, which is essential for the degradation of cellular debris and damaged organelles. Studies have shown that oxidative stress can lead to LMP, which compromises lysosomal integrity and function, ultimately triggering cell death signals (Chen et al., 2022). Furthermore, the presence of ROS can lead to the oxidation of key proteins involved in autophagy, such as Beclin-1, and disrupt the autophagic flux, thereby exacerbating cellular damage and contributing to the progression of various NDs (Ning et al., 2022).

**FIGURE 2:**
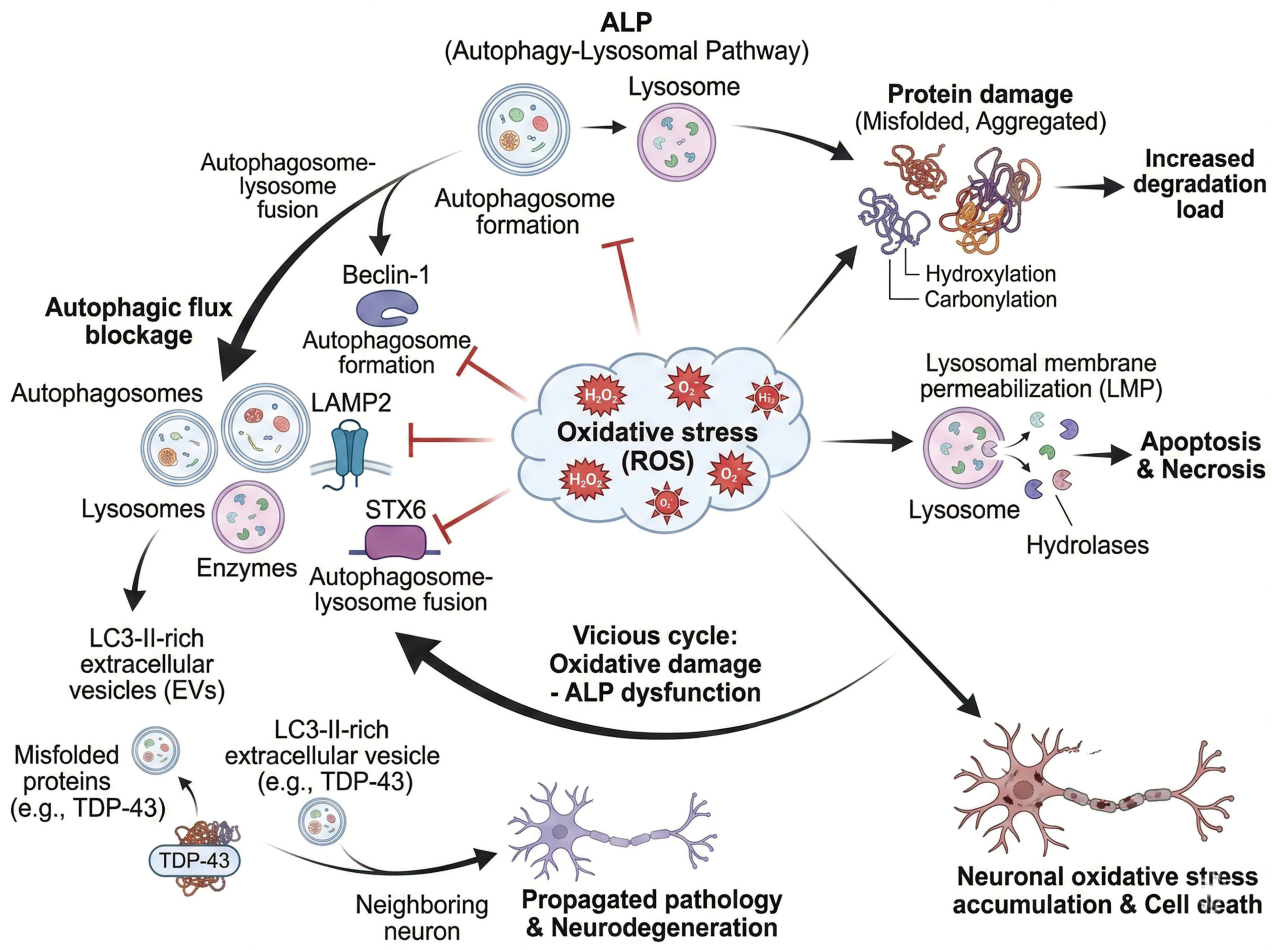
Vicious cycle between oxidative stress and autophagy-lysosomal pathway (ALP) dysfunction in neurodegenerative diseases (NDs). Excessive reactive oxygen species (ROS) impair key molecules of ALP (Beclin-1, LAMP2, STX6), leading to autophagosome formation defect, fusion blockade, and lysosomal membrane permeabilization (LMP). ALP dysfunction further aggravates protein damage and ROS accumulation, forming a vicious cycle. Impaired ALP also promotes the release of LC3-II-rich extracellular vesicles (EVs) containing pathological proteins (e.g., TDP-43), mediating pathological propagation and neuronal death.

Key molecules, such as syntaxin 6 (STX6), play a pivotal role in regulating the release of LC3-II from EVs, which is critical for autophagosome formation. Under oxidative stress conditions, the functionality of STX6 is impaired, leading to an exacerbation of autophagic dysfunction (Vietri et al., 2025). This dysregulation can result in the accumulation of damaged proteins and organelles, further promoting oxidative stress and creating a vicious cycle that is detrimental to cellular health. The interplay between oxidative stress and autophagy is complex; while autophagy serves as a protective mechanism against oxidative damage by degrading oxidized proteins and dysfunctional organelles, excessive oxidative stress can overwhelm this protective response, leading to cellular dysfunction and death (Figure 2; Elbialy, 2020).

In summary, oxidative stress significantly disrupts ALP by impairing autophagosome formation, lysosomal function, and the fusion of these two critical cellular structures. This disruption not only hinders the degradation of damaged cellular components but also promotes a cycle of oxidative damage and cellular dysfunction, highlighting the importance of targeting oxidative stress in therapeutic strategies aimed at restoring autophagic function and mitigating the effects of NDs. Understanding the molecular mechanisms by which oxidative stress influences autophagy is essential for developing effective interventions that can enhance autophagic flux and improve cellular resilience against oxidative damage (Desideri et al., 2025). The field has disproportionately emphasized ROS as damaging agents, while overlooking the potential adaptive role of mild oxidative stress in preconditioning lysosomal function. We propose that transient, controlled activation of oxidative stress pathways might enhance autophagic flux through hormetic mechanisms—a hypothesis that remains largely unexplored but could inform novel timing-dependent therapeutic strategies.

### 3.3 Oxidative damage to key proteins in ALP

Autophagy-lysosome pathway plays a pivotal role in cellular homeostasis by facilitating the degradation of damaged proteins and organelles. However, oxidative stress can lead to the modification of autophagy-related proteins and lysosomal membrane proteins, significantly impacting their functionality and stability. Oxidative modifications, such as carbonylation and sulfenylation, can alter the structure of these proteins, thereby impairing their interactions and functions within the autophagy machinery. For instance, studies have shown that oxidative stress can lead to the inactivation of critical autophagy proteins, including Beclin-1 and LC3, which are essential for autophagosome formation and maturation (Elbialy, 2020). Furthermore, oxidative modifications can destabilize lysosomal membrane proteins, such as LAMP2, leading to LMP. This destabilization compromises the lysosomal integrity, resulting in the leakage of lysosomal enzymes into the cytosol, which can trigger cell death pathways (Abokyi et al., 2021). Collectively, these oxidative modifications disrupt the delicate balance of the autophagy-lysosomal system, leading to impaired protein degradation and accumulation of damaged cellular components.

Moreover, the presence of oxidatively modified proteins can lead to protein cross-linking and aggregation, which further obstructs the transport of autophagosomes to lysosomes and their subsequent fusion. The accumulation of these aggregates not only impedes the autophagic flux but also contributes to cellular stress and inflammation, exacerbating the overall dysfunction of ALP (Lv et al., 2025). For example, in models of NDs, the aggregation of misfolded proteins, such as TDP-43, SOD1, and FUS in ALS, has been linked to impaired autophagic degradation and increased oxidative stress (Bozzo et al., 2017; Casiraghi et al., 2025; Suvarna et al., 2022). The inability to clear these aggregates effectively leads to neuronal toxicity and cell death, underscoring the critical role of ALP in maintaining cellular health under oxidative stress conditions.

The overall efficiency of ALP is significantly diminished in the presence of oxidative damage, leading to a cascade of detrimental effects on protein degradation. The accumulation of oxidized proteins and dysfunctional organelles not only contributes to cellular senescence but also plays a crucial role in the pathogenesis of various diseases, including neurodegenerative disorders, metabolic syndromes, and cancer (Desideri et al., 2025). For instance, in the context of diabetic kidney disease, oxidative stress has been shown to impair lysosomal function, leading to the accumulation of damaged proteins and exacerbating renal injury (Chen et al., 2025). This highlights the importance of maintaining the integrity of ALP as a therapeutic target for mitigating oxidative stress-related pathologies.

In conclusion, oxidative damage to key proteins within ALP significantly impairs its functionality, leading to decreased protein degradation and accumulation of damaged cellular components. This dysfunction is a critical factor in the progression of various diseases, emphasizing the need for therapeutic strategies aimed at restoring autophagic flux and enhancing lysosomal function to combat oxidative stress-induced cellular damage. Future research should focus on identifying specific interventions that can mitigate oxidative modifications and restore the integrity of the autophagy-lysosomal system to promote cellular health and resilience against oxidative stress.

### 3.4 Autophagy blockade and the mechanism of pathological protein extracellular release

The fusion of autophagosomes with lysosomes is a critical step in the autophagic process, allowing for the degradation of cellular debris and misfolded proteins. However, when this fusion is impaired, as seen in various NDs, abnormal proteins can escape the degradation pathway and be released into the extracellular space via EVs that are enriched with LC3-II, a marker of autophagosomes. This process has been particularly well-studied in the context of proteins such as TDP-43, which is implicated in ALS and frontotemporal lobar degeneration (FTLD). Research has shown that when autophagy is inhibited, the secretion of TDP-43 through EVs is significantly increased, suggesting that the failure of autophagosome-lysosome fusion leads to the accumulation of TDP-43 in EVs, facilitating its spread between cells (Tanaka et al., 2023). This mechanism not only highlights the importance of autophagy in maintaining protein homeostasis but also underscores how its dysfunction can contribute to the propagation of pathological proteins in neurodegenerative conditions.

In addition to EV-mediated extracellular release of pathological proteins triggered by autophagy blockade, the formation of extracellular abnormal protein plaques in neurodegenerative diseases also involves non-vesicular pathways closely linked to lysosomal dysfunction. In Alzheimer’s disease (AD), extracellular amyloid−β (Aβ) plaque formation arises not only from EV-transported aggregates but also from direct cleavage of amyloid precursor protein (APP) at the neuronal cell surface (Kassir et al., 2025). Sequential processing of APP by β−secretase and γ−secretase liberates toxic Aβ fragments, which rapidly coalesce in the extracellular space to form insoluble amyloid deposits (Kassir et al., 2025; Strope and Wilkins, 2023). Importantly, this Aβ aggregation process and the generation of PANTHOS (proteopathy-associated thioflavin-S-positive histone-positive) aggregated structures in AD are directly coupled to lysosomal pH impairment (Handy et al., 2024). Defective lysosomal acidification blunts the activity of lysosomal hydrolases responsible for APP metabolism and Aβ clearance, thereby amplifying extracellular Aβ deposition and aggregation, and establishing a vicious cycle with lysosomal dysfunction and autophagy blockade (Bouhamdani et al., 2021; Hu et al., 2022; Ishida et al., 2013).

The intercellular transmission of pathological proteins, such as TDP-43, through EVs exacerbates the spread of neurodegeneration, promoting disease progression. The release of these proteins can induce a toxic environment in neighboring cells, leading to further neuronal damage and dysfunction. In the case of α-synuclein, another protein associated with NDs like PD, studies have shown that its release from damaged neurons can activate microglial cells, which in turn can lead to neuroinflammation and exacerbate neuronal injury (Raj et al., 2024). The role of microglia in this context is particularly critical, as they can uptake and process these pathological proteins, but excessive accumulation can lead to their dysfunction and a shift toward a pro-inflammatory phenotype, further propagating the cycle of neurodegeneration (Gao et al., 2023).

At the molecular level, the mechanisms governing the release of these pathological proteins via EVs involve complex interactions among various proteins, including membrane fusion proteins and those involved in the multivesicular body (MVB) pathway. For instance, syntaxin-6 has been implicated in the regulation of LC3-II-positive EV release, indicating that specific SNARE proteins may play a role in the vesicular transport and secretion of autophagic cargo (Ito and Tanaka, 2024). Additionally, the involvement of the endosomal sorting complexes required for transport (ESCRT) machinery in the biogenesis of EVs further emphasizes the intricate regulatory networks that dictate the fate of misfolded proteins in NDs (Li J. et al., 2025).

Moreover, the interplay between autophagy and the secretion of pathological proteins suggests that therapeutic strategies aimed at enhancing autophagic flux could mitigate the pathological spread of these proteins. For instance, pharmacological agents that promote autophagy have shown promise in preclinical models by reducing the levels of toxic proteins and improving neuronal health (Hussain et al., 2025). Thus, understanding the molecular mechanisms underlying autophagy blockade and the subsequent extracellular release of pathological proteins is crucial for developing effective therapeutic strategies against NDs, where the restoration of autophagic function may play a pivotal role in halting disease progression and improving patient outcomes.

### 3.5 The association of protein degradation dysfunction with neuronal functional decline

The dysregulation of the UPS and ALP can cause the build-up of toxic proteins such as tau and alpha-synuclein, which are hallmarks of NDs like Alzheimer’s and PD (Tang et al., 2019). This accumulation not only disrupts cellular homeostasis but also triggers neuroinflammatory responses, exacerbating neuronal damage. The presence of these toxic proteins can activate various signaling pathways that promote inflammation and cell death, further impairing neuronal function and survival (Pytel and Fromm Longo, 2025). Thus, the relationship between protein degradation dysfunction and neuronal decline is complex and multifaceted, with the accumulation of toxic proteins serving as a central mechanism through which neuronal health is compromised.

Moreover, the imbalance of intracellular protein homeostasis can lead to the activation of inflammatory responses that further aggravate neuronal damage. When protein degradation pathways are compromised, the resultant accumulation of misfolded proteins can activate microglia and astrocytes, leading to a pro-inflammatory environment that exacerbates neuronal injury (Wu et al., 2024). This inflammatory response is characterized by the release of cytokines and ROS, which can induce oxidative stress and further damage neuronal cells. The interplay between protein aggregation, inflammation, and neuronal dysfunction highlights the critical need for effective protein quality control mechanisms to maintain neuronal health. In NDs, the chronic activation of these inflammatory pathways due to persistent protein accumulation can serve as a significant driver of disease progression (Dou et al., 2021). Therefore, the dysregulation of protein degradation not only affects the immediate cellular environment but also has far-reaching implications for neuronal survival and function.

Long-term dysfunction in protein degradation pathways is emerging as a pivotal factor in the pathological progression of NDs. Studies indicate that chronic impairment of the UPS and autophagy leads to a vicious cycle of protein accumulation and neuronal decline, where the inability to clear damaged proteins contributes to cellular senescence and apoptosis (Raj et al., 2024). This progressive decline in cellular function is particularly evident in aging neurons, which are already predisposed to stress due to their post-mitotic nature and high metabolic demands. As NDs progress, the efficiency of protein degradation mechanisms continues to diminish, leading to an exacerbation of neuronal dysfunction and eventual cell death (Xu et al., 2023). Thus, addressing protein degradation dysfunction presents a promising therapeutic target for mitigating neuronal decline and improving outcomes in NDs.

## 4 LQC mechanism and its role in NDs

Mounting evidence has established that lysosomal dysfunction is a convergent pathological feature across multiple neurodegenerative disorders, including AD, PD, amyotrophic lateral sclerosis, and Huntington’s disease. To counteract lysosomal damage and preserve organellar function, cells have evolved a sophisticated and multilayered LQC system, which encompasses lysosomal membrane repair, selective removal of impaired lysosomes via lysophagy, *de novo* lysosomal biogenesis and regeneration, as well as fine-tuned signaling regulation. Defects in any step of the LQC cascade can trigger a vicious cycle of lysosomal impairment, proteotoxic stress, neuroinflammation, and progressive neuronal degeneration. In this section, we systematically summarize the core molecular machineries underlying LQC, highlight the crosstalk between its distinct sub-pathways, and discuss the critical contributions of LQC dysregulation to the onset and progression of NDs, aiming to provide mechanistic insights for the development of novel therapeutic strategies targeting lysosomal homeostasis.

### 4.1 Mechanisms of lysosomal repair

When lysosomal membranes are damaged, it triggers a rapid repair process aimed at restoring membrane integrity and enzymatic activity. This repair mechanism is essential for preventing cell death and ensuring neuronal survival, particularly in the context of NDs. Lysosomal membrane damage triggers either ESCRT-dependent or ESCRT-independent lysosomal membrane repair. Upon lysosomal injury, the ESCRT complex is rapidly recruited through pathways such as LRRK2-RAB8A (Herbst et al., 2020) and PDCD6-ALIX-TSG101 (Shukla et al., 2022) to initiate membrane repair. However, the specific ESCRT components involved in this process, as well as the differences between ESCRT-mediated intraluminal vesicle formation and lysosomal membrane repair, remain to be further elucidated. Loss of ESCRT machinery delays lysosomal membrane repair; nevertheless, repair is not completely abolished. Recent studies highlight the importance of membrane fusion proteins, lipid remodeling, and membrane protein recycling in the lysosomal repair process. For instance, upon LMP, cells activate various membrane remodeling processes, including membrane invagination, tubulation, and lipid patching, which are crucial for restoring lysosomal integrity (Xun and Tan, 2025). The phosphoinositide-initiated membrane tethering and lipid transport (PITT) pathway has been identified as a key mechanism that facilitates rapid lysosomal repair by enabling the transfer of lipids from the endoplasmic reticulum (ER) to damaged lysosomes (Heinke, 2022). This process is orchestrated by proteins such as oxysterol-binding protein (OSBP) and the lipid transfer protein ATG2, which play pivotal roles in lipid transfer and membrane fusion (Goul and Zoncu, 2022).

The maintenance of lysosomal function is essential for preventing cellular stress, inflammation, and ultimately cell death. In the context of NDs, where lysosomal dysfunction is a hallmark, the ability to repair damaged lysosomes becomes even more critical. Studies have shown that aging and neurodegenerative conditions, such as AD, impair lysosomal repair mechanisms, leading to increased cellular vulnerability and neurodegeneration (Bingol, 2018; Chua et al., 2022; Todd et al., 2023; Vrancx and Annaert, 2025; Yu et al., 2018). The accumulation of damaged lysosomes can trigger inflammatory responses, exacerbating neuronal loss and contributing to disease progression (Wang and Baehrecke, 2025). Therefore, understanding the molecular mechanisms underlying lysosomal repair is not only vital for elucidating the pathogenesis of NDs but also for developing therapeutic strategies aimed at enhancing lysosomal function and promoting cellular survival.

Moreover, the interplay between lysosomal repair and autophagy is significant. Autophagy is a cellular process that degrades and recycles damaged organelles and proteins, and it is closely linked to lysosomal function. In cases where lysosomal repair fails, cells may resort to lysophagy, a selective form of autophagy that targets damaged lysosomes for degradation (Zhang R. et al., 2025). This process highlights the cell’s adaptive response to maintain homeostasis and prevent the detrimental effects of lysosomal damage. The involvement of galectins, which recognize glycosylated structures exposed during lysosomal damage, further emphasizes the complexity of the lysosomal repair response (Sudhakar et al., 2020).

In conclusion, the mechanisms of lysosomal repair are multifaceted and involve a coordinated response that includes membrane fusion, lipid transfer, and autophagic processes. These mechanisms are crucial for maintaining lysosomal integrity, which in turn is vital for neuronal survival and overall cellular health. As research continues to uncover the intricacies of lysosomal repair pathways, there is potential for developing targeted therapies that enhance these processes, offering hope for treating NDs characterized by lysosomal dysfunction.

### 4.2 Lysophagy in the clearance of damaged lysosomes

Lysophagy is a selective autophagic process triggered by LMP resulting from oxidative stress or toxic aggregates (Liu et al., 2020). This pathway relies on the recognition of damaged membranes by galectin-3 and subsequent polyubiquitination of lysosomal proteins by E3 ubiquitin ligases (Teranishi et al., 2022). Receptors such as p62/SQSTM1 then recruit autophagic machinery to sequester and degrade the damaged organelles (Gallagher and Holzbaur, 2023).

In the context of NDs, the failure to execute lysophagy is a critical driver of neuronal death (Abokyi et al., 2021). The importance of lysophagy is underscored by the observation that mutations affecting lysophagy components can lead to increased neurodegeneration. For example, in models of ALS, mutations in the p62 gene have been shown to impair lysophagy, resulting in the accumulation of damaged lysosomes and contributing to neuronal death (Klickstein et al., 2024). Moreover, therapeutic strategies aimed at enhancing lysophagy have emerged as potential interventions for neurodegenerative conditions. For instance, compounds that activate lysophagy pathways, such as tetrandrine, have been shown to promote the clearance of damaged lysosomes and restore cellular homeostasis (Yang et al., 2025).

Overall, lysophagy serves as a critical quality control mechanism for lysosomes, ensuring the removal of damaged organelles and preventing the detrimental effects of lysosomal dysfunction. Its dependence on specific receptors and ubiquitin signaling highlights the intricate regulatory networks that govern lysosomal homeostasis. As research continues to unravel the complexities of lysophagy, it holds promise for developing targeted therapies aimed at enhancing lysosomal function in various diseases, particularly neurodegenerative disorders where lysosomal integrity is compromised.

### 4.3 Lysosomal regeneration and functional recovery

The regulation of lysosomal biogenesis is significantly influenced by transcription factors, notably TFEB, which orchestrates the expression of lysosomal genes essential for the synthesis of new lysosomes. TFEB is activated under conditions of nutrient deprivation or stress, leading to enhanced lysosomal function and biogenesis. Studies have shown that TFEB-mediated pathways promote the transcription of genes involved in lysosomal enzyme production, membrane trafficking, and autophagy, thereby facilitating the generation of new lysosomes to replace damaged ones and restore cellular degradation capabilities (Huang et al., 2024). This regenerative capacity is vital, as it helps to maintain the cellular degradation machinery necessary for the clearance of damaged organelles and proteins, which is particularly important in the context of NDs where the accumulation of misfolded proteins can lead to cellular toxicity (Bhattacharya et al., 2023).

The mechanisms underlying lysosomal regeneration are multifaceted and involve the replenishment of damaged lysosomes, which is essential for sustaining cellular degradation capacity. Following lysosomal damage, cells can initiate a regeneration process that includes the removal of dysfunctional lysosomes through lysophagy, a selective autophagic process. This not only clears damaged organelles but also allows for the recycling of lysosomal components, thus supporting the formation of new lysosomes (Lancaster et al., 2021). Impaired lysosomal function triggers cellular compensatory repair and regeneration programs, under mild stress conditions, cells activate TFEB/TFE3-mediated lysosomal biogenesis through the CASM pathway (Xu et al., 2019) and the AMPK-FNIP1 axis (Malik et al., 2023). In contrast, under severe membrane damage, MCOLN1-mediated calcium release (Hooper et al., 2022; Medina et al., 2015) and the LGALS3-SMURF1-PPP3 signaling axis (Xia et al., 2024, 2025) further amplify TFEB activation, simultaneously initiating lysosomal regeneration and autophagy to restore lysosomal degradative function and maintain cellular homeostasis.

Moreover, recent research has highlighted the importance of proteins such as TBC1D15 in the regeneration of lysosomes after damage. TBC1D15 is involved in the recruitment of autophagic machinery to damaged lysosomes, facilitating membrane repair and the formation of new lysosomal tubules, which are crucial for restoring lysosomal function (Shi et al., 2020). This regenerative process is not merely a response to damage but is also a proactive mechanism that ensures cellular resilience against stressors that could compromise lysosomal integrity.

The pathological implications of impaired lysosomal regeneration are particularly evident in NDs, where lysosomal dysfunction is closely linked to disease progression. For instance, in AD, the failure of lysosomal regeneration contributes to the accumulation of amyloid plaques and tau tangles, which exacerbate neuronal damage and cognitive decline (Sava et al., 2024). This highlights the critical need for therapeutic strategies aimed at enhancing lysosomal function and regeneration. Recent studies have explored the potential of pharmacological agents and gene therapies to activate TFEB and promote lysosomal biogenesis, offering promising avenues for intervention in diseases characterized by lysosomal dysfunction (Li Y. Y. et al., 2025). Additionally, approaches that enhance the autophagic-lysosomal pathway, such as the use of small molecules that stimulate autophagy, have shown potential in restoring lysosomal function and improving cellular health in neurodegenerative contexts (Vantaggiato et al., 2025).

In conclusion, lysosomal regeneration and functional recovery are essential processes for cellular health, particularly in the context of NDs. The regulation of lysosomal biogenesis through transcription factors like TFEB, coupled with the mechanisms of lysophagy and the involvement of specific proteins in lysosomal repair, underscores the complexity of lysosomal dynamics in maintaining cellular homeostasis. Understanding these processes opens new avenues for therapeutic interventions aimed at enhancing lysosomal function, which could mitigate the progression of NDs and improve patient outcomes.

### 4.4 Regulatory network and signaling pathways of LQC mechanism

**FIGURE 3:**
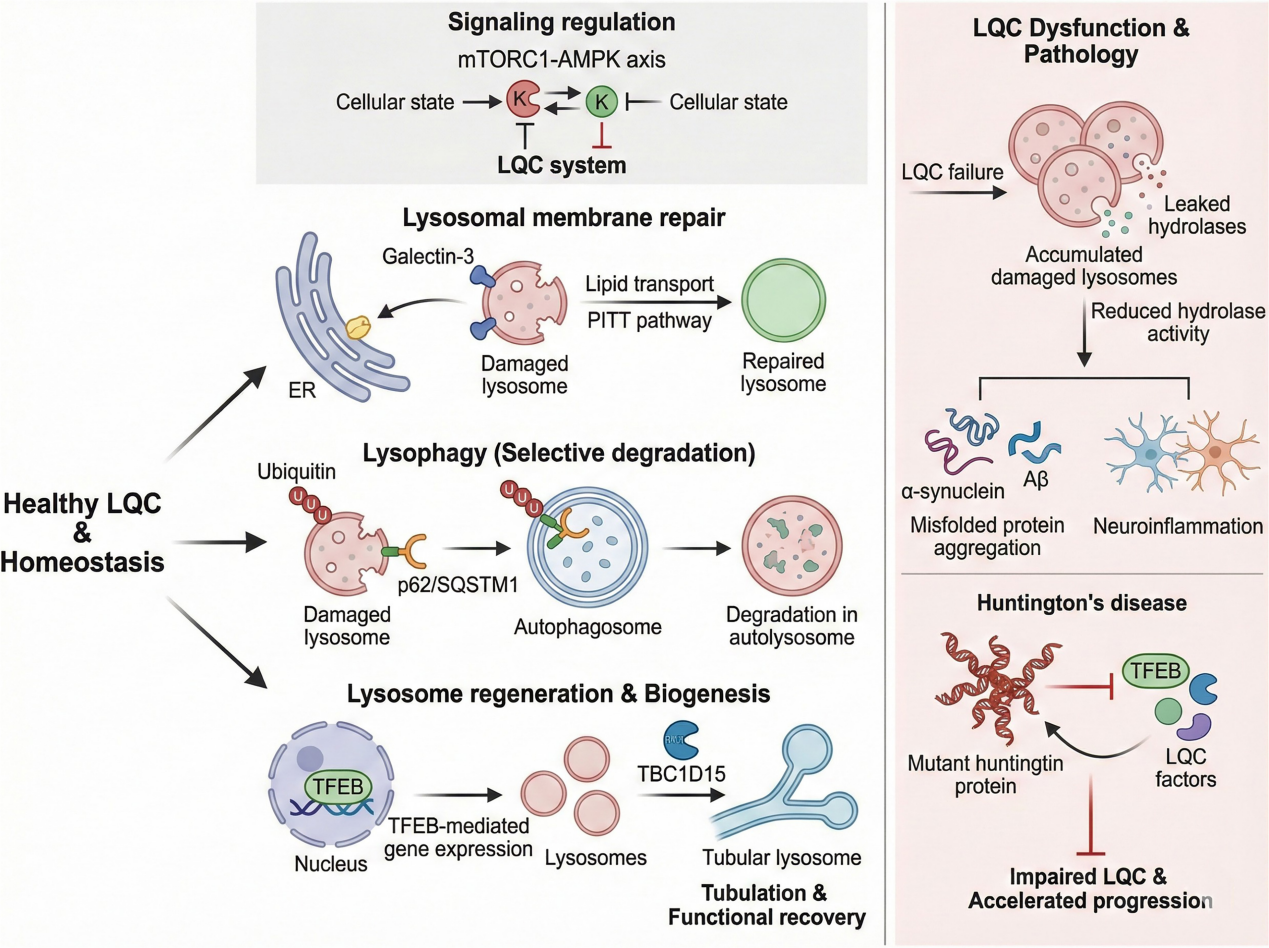
Schematic diagram of the lysosomal quality control (LQC) system and its dysregulation in neurodegenerative diseases (NDs). Under physiological conditions, the LQC system maintains lysosomal homeostasis through three interconnected sub-pathways: lysosomal membrane repair (mediated by the PITT pathway and galectin-3), selective degradation of damaged lysosomes via lysophagy (mediated by p62/SQSTM1), and lysosomal regeneration/biogenesis (regulated by TFEB and TBC1D15). The mTORC1-AMPK axis serves as the central signaling hub to fine-tune LQC activity in response to cellular states. In NDs (e.g., Huntington’s disease), LQC dysfunction leads to the accumulation of damaged lysosomes, reduced hydrolase activity, and misfolded protein aggregation, which exacerbates neuroinflammation and accelerates disease progression.

We have classified different lysosomal quality control pathways as independent processes, but in fact they are interrelated. The LQC mechanism is intricately regulated by various signaling pathways, notably the mTORC1 and AMPK pathways, which play crucial roles in maintaining lysosomal function and autophagic activity to achieve cellular metabolic balance. mTORC1 is a key regulator of cell growth and metabolism, responding to nutrient availability and energy status. When nutrients are abundant, mTORC1 is activated, promoting anabolic processes while inhibiting autophagy. Conversely, during nutrient deprivation or stress, mTORC1 activity is suppressed, leading to the activation of autophagy, which facilitates the degradation of damaged organelles and proteins, including dysfunctional lysosomes. AMPK, on the other hand, acts as an energy sensor, becoming activated in response to low energy levels. Upon activation, AMPK promotes catabolic processes, including autophagy, while inhibiting mTORC1 signaling. This interplay between mTORC1 and AMPK is vital for maintaining lysosomal integrity and function, as it ensures that cells can adapt their metabolic processes in response to changing environmental conditions. Dysregulation of these pathways can lead to impaired lysosomal function, contributing to the pathogenesis of various diseases, including neurodegenerative disorders, where lysosomal dysfunction is a common feature (Figure 3; Zhu et al., 2020).

Under cellular stress conditions, the LQC mechanism is activated to counteract lysosomal damage and maintain cellular homeostasis. Stressors such as oxidative stress, nutrient deprivation, and proteotoxicity can lead to LMP, resulting in the leakage of lysosomal enzymes into the cytosol, which can trigger cell death pathways. In response to such damage, LQC initiates a series of protective mechanisms, including lysosomal repair, degradation of damaged lysosomes through autophagy (lysophagy), and the biogenesis of new lysosomes. The activation of LQC is crucial for cellular survival, as it helps to restore lysosomal function and prevent cellular toxicity. For instance, in the context of Huntington’s disease, the accumulation of misfolded proteins can lead to LMP, which activates the LQC system to mitigate damage. However, if the LQC response is insufficient or impaired, it can exacerbate lysosomal dysfunction and contribute to the progression of NDs (NDs) (Rusmini et al., 2025).

Disruption of signaling pathways involved in LQC can lead to a decline in its functionality, thereby promoting the onset and progression of various diseases. For example, in NDs, the impairment of lysosomal function and the subsequent failure of LQC mechanisms can result in the accumulation of damaged organelles and misfolded proteins, which are toxic to cells. This is particularly evident in conditions such as Alzheimer’s and Parkinson’s disease, where lysosomal dysfunction has been linked to increased neurodegeneration. The sequestration of transcription factors like TFEB and TFE3, which are critical for the activation of lysosomal biogenesis and autophagy, into protein aggregates further exacerbates this dysfunction. The loss of these transcription factors impairs the cellular response to lysosomal damage, leading to a vicious cycle of lysosomal impairment and cellular toxicity. Thus, understanding the regulatory networks and signaling pathways that govern LQC is essential for developing therapeutic strategies aimed at restoring lysosomal function and mitigating the effects of NDs (Ferrari et al., 2024).

### 4.5 The pathological significance of LQC in NDs

The dysregulation of LQC is closely associated with lysosomal dysfunction and protein degradation defects, which are critical factors contributing to neuronal damage. In neurodegenerative conditions such as Huntington’s disease (HD), the accumulation of misfolded proteins, particularly the mutant Huntingtin protein (muHTT), leads to the sequestration of essential proteins involved in cellular homeostasis, including transcription factors like TFEB and TFE3. These transcription factors are pivotal for the activation of LQC mechanisms, which include lysophagy and lysosomal biogenesis. When muHTT aggregates sequester these factors, the activation of LQC is hindered, resulting in LMP and the subsequent leakage of hydrolases into the cytosol, which exacerbates cellular toxicity and neuronal death (Rusmini et al., 2025). Furthermore, the disruption of LQC mechanisms can lead to the persistence of damaged lysosomes, creating a vicious cycle that promotes neurodegeneration. This highlights the importance of a balanced LQC system in preventing neuronal injury and maintaining cell viability, as the failure to repair or replace dysfunctional lysosomes can have dire consequences for neuronal health.

Enhancing the functionality of LQC presents a promising therapeutic strategy to potentially delay or halt the progression of NDs. By targeting the pathways involved in LQC, researchers aim to restore lysosomal function and improve the clearance of toxic aggregates. For instance, the overexpression of TFEB and TFE3 has been shown to reduce muHTT aggregation and promote the degradation of damaged lysosomes through lysophagy (Rusmini et al., 2025). This suggests that pharmacological or genetic approaches aimed at enhancing LQC could mitigate the deleterious effects of protein aggregation in NDs. Additionally, restoring LQC may not only improve lysosomal function but could also enhance overall neuronal resilience against various forms of stress, thereby potentially slowing down disease progression. As such, LQC is emerging as a critical target for therapeutic intervention, providing a novel avenue for the treatment of neurodegenerative disorders characterized by lysosomal dysfunction.

The growing body of evidence underscores the significance of LQC as an emerging therapeutic target in the context of NDs. As researchers continue to elucidate the molecular mechanisms underlying LQC and its role in neuronal health, it becomes increasingly clear that restoring lysosomal function through LQC modulation could represent a transformative approach to treating these debilitating conditions. The potential for LQC to serve as a target for therapeutic strategies is supported by findings that demonstrate its involvement in various neurodegenerative processes, including the regulation of autophagy and the clearance of protein aggregates (Ferrari et al., 2024). By harnessing the power of LQC, it may be possible to develop innovative treatments that not only address the symptoms of NDs but also tackle their underlying causes, ultimately improving patient outcomes and quality of life. In conclusion, the pathological significance of LQC in NDs cannot be overstated, as it represents a critical intersection between cellular homeostasis and disease progression, making it a focal point for future research and therapeutic development. Importantly, we note that most LQC studies have been conducted in rapidly dividing cells or acute injury models. Neurons, being post-mitotic and highly polarized, may rely on distinct LQC mechanisms or exhibit different thresholds for activating lysophagy versus lysosomal repair. We suggest that future research should prioritize neuron-specific LQC pathways, as therapeutic strategies derived from non-neuronal systems may not directly translate to the central nervous system.

### 4.6 Neurodegeneration-associated genes in ALP and LQC dysfunction

Beyond the core LQC machinery, several neurodegeneration-associated genes directly regulate ALP integrity and lysosomal function, particularly TDP-43, PGRN, and TMEM106B in ALS and FTD. TDP-43 pathology impairs autophagosome-lysosome fusion and exacerbates lysosomal membrane permeabilization (Meneses et al., 2021). PGRN encodes a lysosomal glycoprotein essential for acidification and proteolysis; GRN loss-of-function mutations lead to damaged lysosome accumulation and impaired autophagic clearance (Jing et al., 2016). TMEM106B, a lysosomal transmembrane protein that regulates morphology, pH homeostasis, and trafficking, has emerged as a major genetic modifier of FTD and ALS; its variants interact with the V-ATPase complex, and dysfunction disrupts autophagic flux and endolysosomal pathways (Beel et al., 2018; Perneel et al., 2025; Terryn et al., 2021; Zhong et al., 2025). Together, these three genes exemplify how genetic predispositions converge on ALP and LQC dysfunction, highlighting novel therapeutic targets for ALS, FTD, and related disorders.

## 5 Therapeutic strategies and drug development progress in lysosomal repair

Lysosomal dysfunction and impaired autophagic flux are tightly implicated in the pathological progression of multiple human diseases, especially neurodegenerative disorders, lysosomal storage diseases, and age-related metabolic impairments. Over recent decades, extensive efforts have been devoted to exploring effective strategies aimed at restoring lysosomal integrity, enhancing lysosomal function, and reactivating autophagic activity. These strategies span a broad spectrum, ranging from small molecule pharmacology, antioxidant intervention, and gene-based therapy to extracellular vesicle-mediated delivery and multidisciplinary combinatorial regimens. The mechanisms, target diseases, and developmental stages of these representative strategies are summarized in Table 1. In this section, we systematically summarize the recent advances in therapeutic strategies and drug development focused on lysosomal repair, highlight representative mechanisms and preclinical or clinical progress, and discuss the translational potential and challenges of these approaches for future disease management.

**TABLE 1:** Therapeutic strategies for restoring autophagy-lysosomal function in neurodegenerative disorders.

Compound name	Mechanism of action	The specific neurodegenerative disease	Stage of development	References/clinical trial registration number
Metformin	mTOR inhibition, AMPK activation	AD	Clinical phase II/III stage	Koenig et al., 2017
		PD	Clinical phase II stage	NCT05781711
		HD	Clinical phase III stage	NCT04826692
		ALS	Clinical phase I/II stage	NCT04121468, NCT06463743, NCT05893225, NCT06812585, NCT05131828
Rapamycin	mTOR inhibition	AD	Clinical phase I stage	Gonzales et al., 2025; Hou et al., 2023
		ALS	Clinical phase II stage	Mandrioli et al., 2023
		HD	Preclinical study	Bailus et al., 2021
		PD	Preclinical study	Zhang Z. et al., 2025
Ambroxol	Increase the activity of lysosomal enzyme glucose brain enzyme (GCase)	PD (GBA mutation)	Clinical phase II/I stage	Siemeling et al., 2024
Trehalose	Activate TFEB	ALS	Clinical phase II/III stage	HEALEY ALS Platform Trial and HEALEY ALS Platform Trial Study Group, 2025
		Spinocerebellar ataxia type 3 (SCA3)	Clinical phase II stage	Yap et al., 2024
Nilotinib	The cellular abelson tyrosine kinase (C-ABL) inhibition	PD	Clinical phase II stage	Pagan et al., 2016; Pagan et al., 2021
		AD	Clinical phase II stage	Pagan et al., 2021
Celastrol	TFEB activation	AD	Preclinical study	Zhang et al., 2022
Resveratrol	AMPK activation	AD	Clinical phase II stage	Moussa et al., 2017
	Nrf2 activation	PD	Preclinical study	Zamanian et al., 2023
LM11A-31	Regulate the p75^NTR^ receptor	AD	Clinical phase II stage	Shanks et al., 2024; Simmons et al., 2025
		PD	Preclinical study	Pensabene et al., 2025
	Upregulate the AKT signal, inhibit the JNK kinase	HD	Clinical phase I stage	Simmons et al., 2016
RH1115	Targeting lamin A/C and LAMP1	AD	Preclinical study	Hippman et al., 2023
AAV-CRISPR/Cas9 targets ZKSCAN3	Knock out ZKSCAN3	HD	Preclinical study	Park et al., 2025

### 5.1 Small molecule drugs promote lysosomal repair and autophagy activation

The role of small molecules in promoting lysosomal repair and activating autophagy has emerged as a promising therapeutic strategy in the treatment of NDs. Compounds such as TFEB activators and mTOR inhibitors have been identified for their ability to enhance lysosomal biogenesis and autophagic activity, addressing the critical dysfunctions associated with these diseases. TFEB, or transcription factor EB, is a master regulator of lysosomal biogenesis and autophagy, and its activation leads to the transcription of genes essential for lysosome function and autophagic flux. For instance, small molecules like celastrol have been shown to activate TFEB, thereby enhancing autophagic degradation of toxic protein aggregates such as phosphorylated tau in AD models (Zhang et al., 2022). Similarly, mTOR inhibitors, which inhibit the mechanistic target of rapamycin (mTOR) signaling pathway, promote autophagy by alleviating the inhibitory effects of mTOR on autophagic processes. This pathway is particularly relevant as mTOR is known to regulate cellular responses to stress and nutrient availability, and its inhibition has been associated with improved autophagic flux and lysosomal function (Fan et al., 2023).

The therapeutic potential of these small molecules has been demonstrated in preclinical studies, showcasing their ability to restore lysosomal function and enhance protein degradation. For example, the small molecule LM11A-31 has been shown to engage ALP, effectively reducing mutant huntingtin aggregates in models of Huntington’s disease (Simmons et al., 2025). This compound not only promotes the clearance of toxic aggregates but also enhances autophagic activity, highlighting the dual role that such small molecules can play in neuroprotection. Furthermore, the discovery of small molecules that can modulate ALP has opened new avenues for treating lysosomal storage disorders and other neurodegenerative conditions characterized by protein aggregation and impaired autophagy.

In addition to enhancing lysosomal biogenesis, small molecules can also restore lysosomal function by modulating signaling pathways that govern lysosomal activity. For instance, the activation of the TFEB pathway not only promotes the formation of new lysosomes but also enhances the fusion of autophagosomes with lysosomes, a critical step in the autophagic degradation process (Xu et al., 2025). By addressing the underlying mechanisms of lysosomal dysfunction, these therapeutic strategies aim to improve cellular homeostasis and mitigate the pathological features of NDs.

Moreover, the preclinical efficacy of these small molecules has been supported by various studies that demonstrate their potential to improve cognitive function and reduce neurodegeneration in animal models. For example, compounds that enhance autophagic flux have been shown to improve memory deficits in AD models, suggesting that the restoration of autophagic processes can have significant neuroprotective effects (Alhowyan and Harisa, 2025). As research continues to uncover the complex interactions between autophagy, lysosomal function, and neurodegenerative disease pathology, the development of small molecule therapeutics targeting these pathways holds great promise for future clinical applications.

The timing window for small molecule therapy is also crucial. In the early and middle stages of the disease, when protein aggregates are not excessive, not too large in volume, not overly toxic, and the lysosomes still have sufficient degradation capacity and complete quality control mechanisms, small molecules targeting the autophagy-lysosome pathway (ALP) and lysosomal function may have the best therapeutic effect (Siddiqi et al., 2019). Once the protein aggregate load and lysosomal damage exceed the functional limit of the lysosomal quality control system, the damaged lysosomes will be unable to be effectively repaired, recycled or regenerated (Majumder et al., 2011). However, the specific time points for early, middle and late stages may require further exploration, such as finding biomarkers to detect the lysosomal function status. The use of small molecule doses also needs further investigation to ensure safety and efficacy.

In many cases, neurodegenerative disorders are the manifestation of differential abnormalities in signaling pathways and different aspects autophagy lysosomal Pathways. It is hard to pinpoint the exact reason or the order of defects in pathways. In that case combinatorial therapies may be more effective than single small molecule application, without crossing the toxicity threshold. The effectiveness of combined therapy in models of neurodegenerative diseases and clinical studies has been confirmed by numerous studies. For instance, a large-scale drug combination screening conducted in the fruit fly model of Huntington’s disease demonstrated that a low-dose combination of drugs targeting multiple targets such as the autophagy-lysosome pathway, mitochondrial function, and inflammatory response could effectively delay the progression of neurodegeneration, and its overall toxicity was significantly lower than that of high-dose single-drug treatment (Agrawal et al., 2005). In mouse models of Alzheimer’s disease and Parkinson’s disease, intranasal combined administration of rifampicin (anti-aggregation) and resveratrol (anti-inflammatory and autophagy enhancer) showed synergistic effects in improving cognitive function and reducing oligomer aggregation, and resveratrol could also counteract the potential hepatotoxicity of rifampicin by increasing the level of brain-derived neurotrophic factor (BDNF) (Umeda et al., 2021).

In conclusion, small molecules that promote lysosomal repair and activate autophagy represent a novel and promising approach to treating NDs. By enhancing lysosomal function and autophagic activity, these compounds can potentially ameliorate the cellular dysfunctions that characterize these conditions, paving the way for innovative therapeutic strategies that could significantly improve patient outcomes. The ongoing exploration of these small molecules in clinical settings will be crucial for determining their efficacy and safety in human populations, ultimately contributing to the advancement of neurodegenerative disease therapies.

### 5.2 Antioxidants and the protein degradation system protection

Antioxidants play a crucial role in mitigating oxidative damage to proteins, thereby alleviating the burden on cellular degradation systems. Oxidative stress is a significant contributor to protein modification, leading to misfolding and aggregation, which can overwhelm the UPS and autophagy pathways responsible for protein degradation. By neutralizing ROS, antioxidants reduce the incidence of protein oxidation and subsequent degradation. For instance, studies have shown that antioxidants like proanthocyanidins activate the Nrf2/ARE signaling pathway, enhancing the expression of genes involved in antioxidant defense and reducing the ubiquitination of oxidatively damaged proteins, thus promoting their stability and functional integrity (Shuhua et al., 2022). This reduction in protein oxidation not only preserves cellular function but also lessens the load on degradation pathways, allowing them to function more efficiently. Moreover, the protective effects of antioxidants extend to key proteins within ALP, ensuring that these systems remain operational even under oxidative stress conditions.

In addition to reducing oxidative damage, antioxidants also help maintain the normal function of essential proteins within ALP. P62/SQSTM1 can help cells cope with oxidative stress by activating the Nrf2 pathway (Hseu et al., 2021). Studies have shown that Pterostilbene can enhance autophagy and exert antioxidant effects by activating the P62/SQSTM1 pathway (Hseu et al., 2021). Furthermore, in conditions such as AD, where oxidative stress and protein aggregation are prominent, antioxidants have demonstrated the ability to reduce neuroinflammation and restore mitochondrial function, which is closely linked to autophagic activity (Singh et al., 2025). This dual action of antioxidants not only protects proteins from oxidative damage but also supports the cellular degradation systems that are vital for maintaining proteostasis.

The potential of combined antioxidant therapies to enhance lysosomal repair efficiency is also a promising avenue for therapeutic intervention. By integrating antioxidants with strategies that target protein degradation systems, such as PROTACs, researchers are exploring multi-modal approaches to treat NDs (Singh et al., 2025). This combination could simultaneously reduce oxidative stress while promoting the selective degradation of toxic protein aggregates, thereby addressing the complex pathology of diseases like Alzheimer’s and Parkinson’s. The synergistic effects of antioxidants in conjunction with targeted degradation strategies highlight a novel therapeutic paradigm that could significantly improve outcomes for patients suffering from neurodegenerative conditions characterized by oxidative stress and impaired protein degradation systems.

In conclusion, antioxidants serve as critical protectors of protein integrity by reducing oxidative damage and supporting the functionality of protein degradation systems. Their ability to activate protective signaling pathways not only enhances the resilience of cellular components against oxidative stress but also ensures the efficient operation of autophagy and proteasomal degradation mechanisms. Future research should focus on elucidating the intricate interactions between antioxidants and degradation systems, as well as developing therapeutic strategies that harness these relationships to combat NDs effectively.

### 5.3 Gene therapy and molecular targeting strategies

Gene therapy represents a transformative approach in the treatment of NDs, particularly in targeting the underlying genetic and molecular dysfunctions that contribute to these complex disorders. One of the primary strategies involves the targeted modulation of ATGs and lysosomal function genes to restore the activity of the autophagy-lysosome pathway, which is often disrupted in neurodegenerative conditions. For instance, recent advancements in gene therapy have highlighted the potential of using adeno-associated viral vectors to deliver therapeutic genes that can enhance autophagic flux and lysosomal biogenesis (Boyle et al., 2025; Nader et al., 2025). This approach not only aims to clear toxic protein aggregates characteristic of diseases like Alzheimer’s and Parkinson’s but also to improve cellular homeostasis and neuronal survival. The successful application of such strategies is underscored by studies demonstrating that enhancing the expression of key autophagy regulators, such as TFEB (transcription factor EB), can significantly ameliorate the pathology associated with neurodegeneration by promoting the degradation of misfolded proteins and restoring lysosomal function (Perez et al., 2020).

Moreover, gene editing technologies, particularly CRISPR/Cas9, hold promise for correcting hereditary lysosomal dysfunctions that contribute to NDs. For example, the ability to precisely edit genetic mutations in the GBA gene, which encodes for the lysosomal enzyme glucocerebrosidase, has shown potential in addressing Gaucher disease and its associated neurodegenerative manifestations. By restoring the normal function of this enzyme, gene editing can mitigate the accumulation of toxic substrates that lead to neuronal damage. Additionally, the use of CRISPR technology allows for the exploration of gene knockouts or knock-ins that can provide insights into the roles of specific genes in neurodegenerative processes, paving the way for novel therapeutic targets (Perez et al., 2020). This precision in gene therapy not only enhances the efficacy of treatment but also reduces the risk of off-target effects, a significant concern in earlier gene therapy approaches.

The development of molecular targeting drugs aimed at critical regulatory proteins within ALP is another promising avenue in the treatment of NDs. These drugs are designed to selectively modulate the activity of proteins involved in autophagy regulation, thereby enhancing the clearance of pathological aggregates. For instance, small molecules that activate TFEB have been shown to promote autophagy and lysosomal function, leading to improved outcomes in preclinical models of neurodegeneration (Song et al., 2020). The therapeutic potential of these molecular targeting strategies is further supported by evidence suggesting that they can also reduce neuroinflammation, which is a common feature in many NDs (Singh et al., 2024). By focusing on the modulation of specific pathways and proteins, researchers are developing more targeted and effective therapeutic interventions that could significantly alter the disease course in affected individuals.

In conclusion, the integration of gene therapy and molecular targeting strategies presents a multifaceted approach to addressing the challenges posed by NDs. By focusing on the restoration of autophagy-lysosomal function and correcting genetic defects, these innovative therapies hold the potential to not only alleviate symptoms but also modify disease progression at the molecular level. As research continues to evolve, the clinical application of these strategies may provide new hope for patients suffering from these debilitating conditions, ultimately leading to more effective and personalized treatment options (Xu et al., 2024).

### 5.4 Therapeutic potential mediated by EVs

The therapeutic potential of EVs in the context of NDs is an area of growing interest, particularly due to their ability to deliver therapeutic molecules in a targeted manner. EVs, which are nanoscale lipid bilayer-enclosed particles secreted by various cell types, can encapsulate proteins, lipids, and nucleic acids, facilitating intercellular communication and modulating biological responses (Xiao et al., 2021). One of the promising strategies involves utilizing autophagy-related EVs for the targeted delivery of therapeutic agents. By engineering EVs to carry specific therapeutic molecules, researchers can enhance the precision of drug delivery systems, ensuring that the therapeutic agents reach the desired cells while minimizing off-target effects. This approach is particularly relevant in NDs where localized treatment is essential due to the complex pathology and the presence of the blood-brain barrier, which often limits the efficacy of conventional drug therapies (Zhao et al., 2024).

Moreover, EVs can also play a role in regulating the release of pathological proteins associated with NDs. For instance, studies have shown that EVs can facilitate the clearance of misfolded proteins, such as tau and alpha-synuclein, which are implicated in diseases like Alzheimer’s and Parkinson’s (Hu G. et al., 2025). By modulating the release of these pathological proteins through EVs, it may be possible to disrupt the propagation of neurodegenerative processes, effectively blocking the spread of disease-related pathology. This mechanism underscores the dual role of EVs as both mediators of disease progression and potential therapeutic agents that can halt or reverse the pathological spread (Sigdel et al., 2023).

Emerging technologies are also paving the way for innovative therapeutic strategies using EVs in NDs. For example, the development of engineered EVs that can cross the blood-brain barrier and deliver neuroprotective factors or anti-inflammatory agents represents a significant advancement in the field (Hu N. et al., 2025). These engineered EVs can be tailored to enhance their targeting capabilities, ensuring that they bind specifically to neuronal cells or glial cells that require intervention. The versatility of EVs as drug delivery vehicles, combined with their inherent biocompatibility and low immunogenicity, positions them as a promising avenue for future therapeutic interventions in NDs (Mazouzi et al., 2022).

In conclusion, the therapeutic potential of EVs in NDs is multifaceted, encompassing targeted drug delivery, modulation of pathological protein release, and the application of innovative engineering techniques. This emerging field holds promise for developing novel treatment strategies that could significantly improve patient outcomes and address the limitations of current therapeutic approaches. As research continues to elucidate the complex roles of EVs in NDs, it is crucial to explore their full potential in clinical settings, paving the way for effective and personalized therapeutic options (Scuteri and Donzelli, 2024).

### 5.5 Multidisciplinary comprehensive treatment model

**FIGURE 4:**
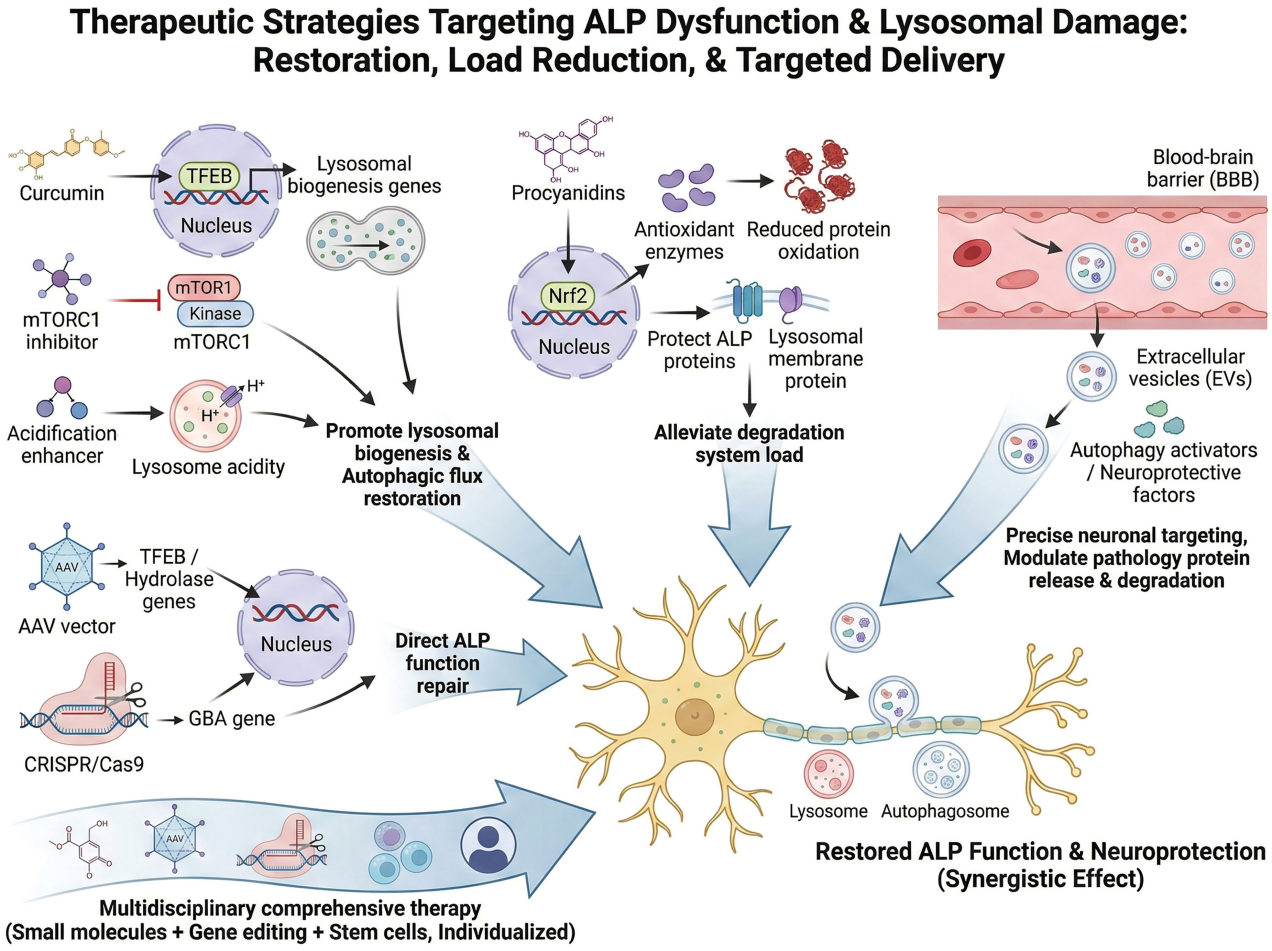
Schematic diagram of therapeutic strategies targeting autophagy-lysosomal pathway (ALP) dysfunction and lysosomal damage in neurodegenerative diseases (NDs). Therapeutic strategies are categorized into four core modules: (1) Small molecule drugs (e.g., TFEB activators, mTOR inhibitors, acidification enhancers) to promote lysosomal biogenesis and autophagic flux restoration; (2) Antioxidants (e.g., procyanidins) to activate the Nrf2 pathway, reduce protein oxidation, and alleviate degradation system load; (3) Gene therapy (e.g., AAV vectors, CRISPR/Cas9) for direct ALP function repair and molecular targeting; (4) Extracellular vesicle (EV)-mediated delivery for precise neuronal targeting and modulation of pathological protein release. These strategies can be integrated into a multidisciplinary comprehensive therapy model (combining small molecules, gene editing, and stem cells) to achieve synergistic neuroprotective effects and restore ALP function.

The integration of pharmacological, genetic, and cellular therapies presents a promising multidimensional approach to restore the functionality of ALP, particularly in the context of NDs. Recent studies have highlighted the critical role of autophagy in maintaining cellular homeostasis and its dysfunction in various neurodegenerative disorders, such as AD and PD (Krogsaeter et al., 2022). Pharmacological interventions, including the use of small molecules that activate autophagy, have shown potential in enhancing the clearance of toxic protein aggregates and dysfunctional organelles (Kang and Kim, 2021). For instance, compounds that target TFEB, a master regulator of ALP, have been identified as promising candidates for therapeutic development (Song et al., 2021). Genetic therapies, such as gene editing techniques, can be employed to correct mutations that impair ATGs, thereby restoring normal cellular functions (Tripathi et al., 2021). Furthermore, cellular therapies, including stem cell transplantation, can provide a source of healthy cells capable of integrating into damaged neural networks and potentially rejuvenating the autophagic processes (Yang et al., 2024). This multifaceted approach not only addresses the symptoms of NDs but also targets the underlying molecular mechanisms, thereby enhancing therapeutic efficacy (Figure 4).

Individualized treatment strategies are essential for optimizing therapeutic outcomes while minimizing adverse effects in patients with NDs. Personalization of treatment regimens can be achieved through the assessment of genetic, epigenetic, and environmental factors that influence disease progression and treatment response (Liu et al., 2025). For example, genetic profiling can identify specific mutations that may affect the efficacy of certain pharmacological agents, allowing for tailored drug selection that aligns with the patient’s unique genetic makeup (Mozolewski et al., 2021). Moreover, biomarkers of autophagy and lysosomal function can be utilized to monitor treatment responses and adjust therapeutic interventions accordingly (Peck et al., 2024). This individualized approach not only enhances the effectiveness of therapies but also reduces the likelihood of adverse effects, as treatments can be fine-tuned to the patient’s specific physiological context (Zhang et al., 2021). By integrating personalized medicine into the treatment paradigm, clinicians can better manage the complexities associated with NDs, ultimately improving patient outcomes.

The comprehensive treatment model facilitates the long-term management and improved prognosis of NDs by fostering collaboration among various disciplines, including neurology, pharmacology, genetics, and rehabilitation. This collaborative approach encourages the sharing of knowledge and resources, enabling the development of innovative therapeutic strategies that address the multifactorial nature of neurodegenerative disorders (Nixon and Rubinsztein, 2024). For instance, interdisciplinary teams can work together to design clinical trials that evaluate the efficacy of combination therapies, such as pairing pharmacological agents with lifestyle interventions like exercise and nutrition, which have been shown to support autophagic processes (Costa et al., 2025). Additionally, by engaging patients and caregivers in the treatment planning process, healthcare providers can ensure that interventions are aligned with patient preferences and values, thereby enhancing adherence and overall satisfaction with care (Ando et al., 2025). Ultimately, this comprehensive and collaborative model not only aims to improve the clinical management of NDs but also seeks to enhance the quality of life for patients and their families, paving the way for more effective and sustainable healthcare solutions.

## 6 Future research directions and challenges

While mechanistic insights into ALP dysfunction have advanced, translating these findings into clinical practice remains hindered by limited biomarkers and complex regulatory networks. This section outlines future research priorities across three key areas: resolving molecular and biomarker gaps, intercepting extracellular protein propagation, and advancing clinical translation through personalized medicine. Addressing these challenges is essential to bridge the gap between laboratory discovery and clinical practice, ultimately facilitating effective neurodegenerative disease management.

### 6.1 Addressing gaps in molecular mechanisms and diagnostic biomarkers

Despite significant progress in understanding ALP dysfunction, several critical challenges remain. First, the identification of sensitive and specific biomarkers is paramount for early diagnosis and monitoring treatment efficacy. Future research should prioritize the validation of cargo proteins within brain-derived extracellular vesicles (EVs)—such as Cathepsin D, LC3-II, and p62—as non-invasive “liquid biopsy” candidates in larger clinical cohorts (Liu W. L. et al., 2022; Snanoudj et al., 2024).

Second, while the PITT pathway and galectin-mediated repair have been identified as core components of lysosomal quality control (LQC), the precise spatiotemporal regulation of these mechanisms under chronic neurodegenerative stress remains poorly understood. High-throughput screening combined with CRISPR/Cas9 technology is needed to identify novel genetic modifiers that can be pharmacologically targeted to enhance these endogenous repair pathways (Ebstrup et al., 2024; Vrancx and Annaert, 2025).

Finally, the dynamic interplay between protein oxidative damage and degradation efficiency requires deeper investigation. Future studies must move beyond static observations to elucidate how specific post-translational modifications, such as the oxidation of p62 or the inactivation of Beclin-1, dictate the “tipping point” from compensated proteostasis to irreversible neuronal death (Luan et al., 2025). Addressing these mechanistic gaps will be essential for developing personalized therapeutic strategies that can be timed to the specific stage of disease progression.

### 6.2 Mechanisms of extracellular propagation of pathological proteins in the brain and interventions

Future research must prioritize intercepting the extracellular propagation of pathological proteins, as this remains a central driver of disease progression. While the roles of the neuronal gene Arc and I-BAR protein IRSp53 in packaging tau into LC3-II-enriched EVs have been identified (Tyagi et al., 2024), a major challenge lies in developing selective inhibitors that can block this pathological release without disrupting essential intercellular communication. Simultaneously, optimizing extracellular waste removal via the glymphatic system represents a critical frontier. Although modulating AQP4 to enhance glymphatic flow shows promise in reducing misfolded aggregates (Lopes et al., 2024), the long-term efficacy and safety of such interventions in human patients require rigorous evaluation. Furthermore, the development of synergistic, multifaceted therapies—integrating tau-targeting antibodies with small-molecule inhibitors—is essential to arrest prion-like propagation at multiple stages (Wu et al., 2025). Elucidating these precise molecular pathways will be vital for translating mechanistic insights into innovative clinical interventions that can effectively halt the spread of proteinopathy.

### 6.3 Clinical translation and development of novel therapeutic approaches

The clinical translation of ALP-targeted therapies represents the next critical frontier, yet several challenges must be overcome to bridge the gap from bench to bedside. Future research should prioritize rigorous clinical evaluation of agents that enhance lysosomal activity or restore autophagic flux (Nixon and Rubinsztein, 2024). Specifically, pharmacological modulators of lysosomal ion channels, such as TRPML1 and TPC2, require systematic safety profiling and efficacy testing to validate their potential in clearing toxic aggregates (Tedeschi et al., 2025). Significant hurdles remain in trial design, including the establishment of standardized inclusion criteria, optimal dosing regimens, and the identification of robust biomarkers to predict treatment response. To address the multifactorial nature of neurodegeneration, future strategies must shift toward multi-target combination therapies—integrating autophagy inducers with lysosomal repair agents—to achieve synergistic neuroprotective effects (Jiao et al., 2024). Furthermore, embracing personalized precision medicine by leveraging genomics, proteomics, and metabolomics will be essential to stratify patient subgroups and tailor therapeutic regimens to individual genetic predispositions and disease phenotypes. Developing this robust, individualized framework is vital for improving the quality of life and clinical outcomes for patients with neurodegenerative disorders.

Beyond current repair mechanisms, we propose a higher-order concept-lysosomal resilience-defined as the ability of neurons to maintain or restore lysosomal function under sustained proteotoxic and oxidative stress. Unlike reversible acute injury, chronic neurodegeneration involves repeated sublethal insults that progressively erode this resilience. How to enhance and quantify this resilience represents a major unmet need, one that could transform preclinical drug discovery and clinical trial design.

## 7 Conclusion

In conclusion, ALP dysfunction is a central driver of ND pathogenesis. This review highlights the critical interplay between oxidative stress and LQC as a pivotal nexus for therapeutic intervention. While emerging small molecules and biological agents show significant promise in restoring lysosomal integrity and autophagic flux in preclinical models, successful clinical translation remains the primary hurdle. Future research must prioritize the identification of sensitive, specific biomarkers and the dissection of disease-specific molecular nodes to facilitate precision medicine. Ultimately, bridging the gap between bench and bedside through multidisciplinary collaboration and integrated, multi-target strategies will be essential to transform the management of NDs from symptomatic relief to fundamental cellular repair.
